# Anti-Inflammatory and Antinociceptive Activities of a Hydroethanolic Extract of *Tamarindus indica* Leaves

**DOI:** 10.3797/scipharm.1110-09

**Published:** 2012-04-01

**Authors:** Santosh Singh Bhadoriya, Vijay Mishra, Sushil Raut, Aditya Ganeshpurkar, Sunil K. Jain

**Affiliations:** 1 Department of Pharmacology, Adina Institute of Pharmaceutical Sciences, Sagar (M.P.) – 470002, India.; 2 Department of Pharmaceutical Sciences, Dr. H. S. Gaur University, Sagar (M.P.) – 470003, India.; 3 Department of Pharmacology, Shri Ram Institute of Technology, Jabalpur (M.P.) – 482002, India.

**Keywords:** *Tamarindus indica*, Carrageenan, Prostaglandins, Membrane stabilization, Flavonoids

## Abstract

The present study aimed to investigate the anti-inflammatory and anti-nociceptive potential of a hydroethanolic extract of *Tamarindus indica* L. leaves (HTI) along with its possible mode of action. The anti-inflammatory activity of HTI was estimated by carrageenan-induced hind paw oedema in male Wistar albino rats. Furthermore, HTI was assessed to determine its effects on membrane stabilization. The antinociceptive action was determined by acetic acid-induced writhing, tail-flick, and the hot plate model. Oral administration of HTI at the dose of 500, 750, and 1000 mg/kg body weight produced significant (*P*< 0.01) anti-inflammatory as well as antinociceptive actions in a dose-dependent manner. Among all tested doses, 1000 mg/kg, *p. o.* reduced carrageenan-induced rat paw oedema at 1, 2, 3, and 4 h. Moreover, the 1000 mg/kg dose exhibited maximum percentage inhibition of acetic acid-induced writhing (48.9%), whereas standard drug diclofenac (25 mg/kg, *p. o.*) showed maximum inhibition (50.9%) of writhing. In the hot plate model, HTI (1000 mg/kg, orally) increased mean basal reaction time after 120 min (7.12±0.05 sec). In the tail flick model, HTI increased the maximum percentage of latency (36.06%), whereas the standard drug pethidine (4 mg/kg, intraperitoneally) showed maximum percentage of latency (43.85%) after 60 min. The findings of the present study supported anti-inflammatory and antinociceptive claims of *T. indica* as were mentioned in Indian traditional and folklore practices.

## Introduction

Inflammation is part of a multifaceted biological phenomenon of vascular tissues to injurious stimuli due to pathogens, injured cells or irritants. It is a defensive attempt by the organism to remove such injurious stimuli and commence the healing process [1], but the symptoms like swelling, tightness, joint pain and irritation associated with inflammation cause patient discomfort. Combating the inflammation can improve circulation and aid healing as well as lessen the pain. Conventional medicines such as steroids and non-steroidal anti-inflammatory drugs (NSAIDs) have shown only limited achievement against all forms of inflammatory circumstances. Furthermore, the unpleasant side effects associated with these drugs such as bleeding and mucosal damage and other gastrointestinal disturbances make the treatment difficult [2]. Considering the probable adverse effects of these drugs, as well as their limited ability to provide long-term remission, there is a need of a new, effective and safe anti-inflammatory agent which can reduce pain and other associated symptoms [3]. To overcome these problems the preparations from plant origin become important in modern medicine and widely prescribed in traditional medicinal systems.

*Tamarindus indica* L. (*Fabaceae*) is extensively used in the traditional system of medicine in India and other countries for treating abdominal pain, diarrhea, wound healing, fever, constipation, blood disorders and acne. The plaster of leaves is applied for curing inflammation locally [4, 5]. Reports of phytochemical investigation of *T. indica* have previously demonstrated the presence of phenolic compounds such as catechin, procyanidin B_2_, epicatechin. Other constituents like tartaric acid, mucilage, pectin, arabinose, xylose, galactose, glucose, uronic acid and triterpenes have also been identified in *T. indica*. [6–8]. An attempt has been made through the membrane stabilizing property of leaves extract to investigate the ability of *T. Indica* in the treatment of inflammatory disorders and its antinociceptive action.

## Results and Discussion

**Fig. 1:**
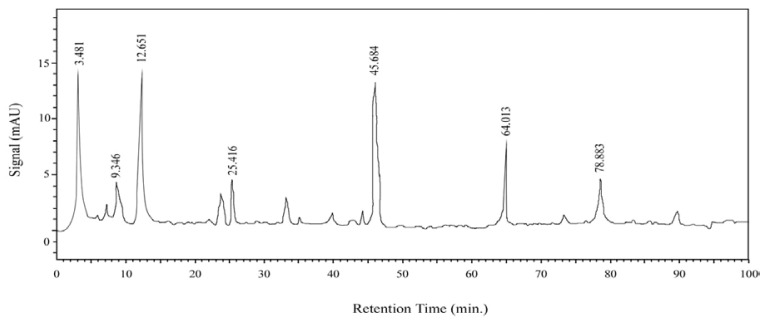
HPLC-UV analysis of the hydroethanolic extract of *T. indica* leaves

As an initial chemical characterization of the hydroethanolic extract of *T. indica* leaves (HTI), a HPLC study was performed. HPLC spectra showed a similar absorption pattern, with two bands of maximum absorption around 230 and 290 nm suggesting the predominance of compounds like flavonoids that normally occur as secondary metabolites in plants (Fig. 1). The HTI showed the presence of flavonoids and polyphenols (Table 1). The results were in accordance with the previously reported data [9].

### Toxicological analysis

As shown in Table 2, the HTI had no lethal or harmful effects on the central as well as autonomous nervous system and motor activity in rats at the tested doses (500–1000 mg/kg body weight, administered *per oral*). The estimated hematological parameters showed no major deviation from the normal group but indicated the increase in the number and volume of blood components. There was a significant rise in the number of WBCs, especially lymphocytes and neutrophils, but the platelets count increased mildly after the seventh day of study (Table 3).

### Anti-inflammatory activity

**Fig. 2:**
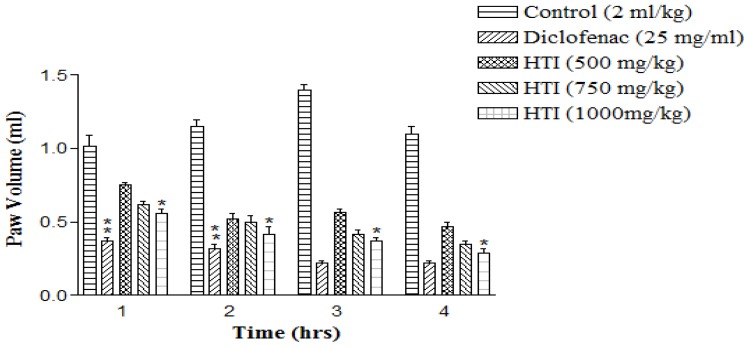
Anti-inflammatory effect of HTI on carrageenan-induced paw oedema in rats. Data represents mean ± SEM (n=5).*P< 0.01; **P< 0.001 as compared with control group.

The effects of HTI on λ-carrageenan-induced oedema in the rat paw are summarized in Fig. 2. The subcutaneous injection of λ-carrageenan into the foot-pad of rats in test and control groups produced a local oedema in the following 1 h that increased progressively to its peak at 3 h and then began to decline. The HTI significantly reduced λ-carrageenan-induced paw edema in a dose-dependent manner at each time point. The standard drug diclofenac also showed an apparent inhibition of the inflammation induced by λ-carrageenan as compared to the control group and was found to be the maximum (84.28%) at 3 h. Among the tested doses of HTI, 1000 mg/kg/po dose showed the maximum inhibitory effect (73.63%) at 3 h, followed by two other doses, 500 mg/kg (59.28%) and 750 mg/kg (70.0%), respectively.

### Antinociceptive activity

**Fig. 3:**
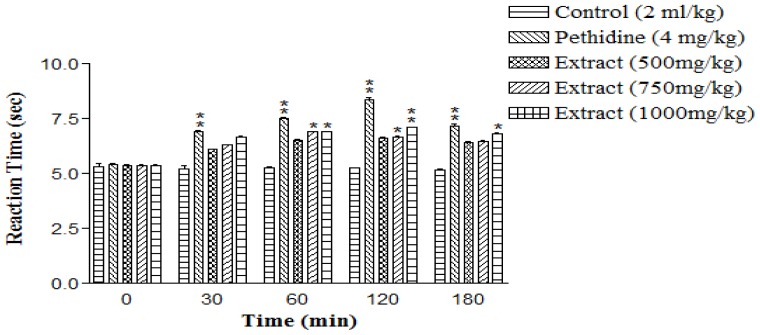
Antinociceptive effect of HTI on hot plate induced algesia in rats. Data represents mean ± SEM (n=5), *P< 0.01; **P< 0.001 compared with control group.

In hot plate induced analgesia, the HTI (750 mg/kg, *per oral*) showed significant (*P< 0.01) increase in mean basal reaction time (6.90±0.02 sec) after 60 min as compared to control (5.29±0.02 sec). The highest nociception inhibition of stimulus exhibited by HTI (1000 mg/kg, *per oral*) was observed after 120 min (7.12±0.05 sec), whereas standard drug pethidine (4 mg/kg, intra peritoneal) showed maximum inhibition (8.36±0.12 sec) after 120 min as compared to control treatment (Fig. 3).

**Fig. 4:**
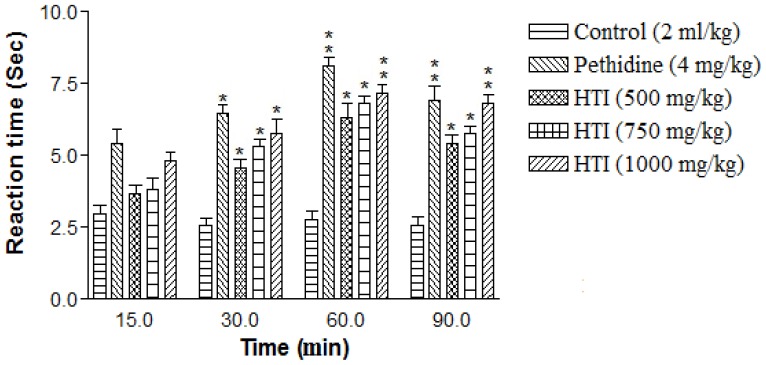
Antinociceptive effect of HTI on tail flick induced algesia in rats. Data represents mean ± SEM (n=5). *P< 0.01; **P< 0.001 compared with control group.

Furthermore, the HTI increased the tail-flick latency of rats toward the thermal stimulus in a dose-dependent manner (Fig. 4). Oral administration of HTI at the dose of 500, 750 and 1000 mg/kg produced the statistically significant (*P< 0.01) antinociceptive action at 60 min. On tested doses of HTI, the increased percent latency was 28.68%, 34.11% and 36.06%, respectively, as compared to the control group. Meanwhile only 1000 mg/kg per oral dose was able to produce the significant antinociceptive action at 90 min, whereas standard drug pethidine (4 mg/kg, intra peritoneal) showed statistically significant (*P< 0.01) results. The percentage of latency was found to be 31.21%, 43.85% and 35.08% at 30, 60 and 90 min time interval, respectively.

### Acetic acid-induced writhing response

**Fig. 5:**
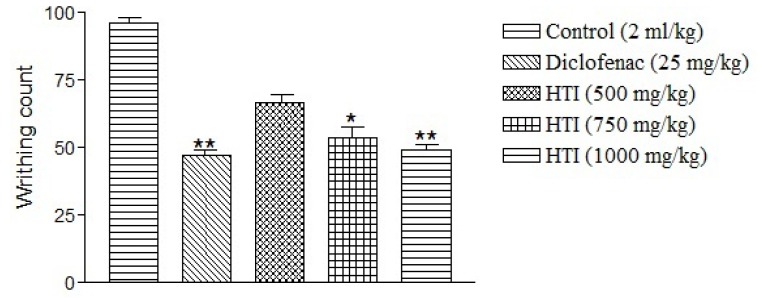
Effect of HTI on acetic acid-induced writhing response in rat. Data represents mean ± SEM (n=5).*P< 0.01; **P< 0.01 as compared with control.

The HTI (500–1000 mg/kg, *per oral*) produced a dose-dependent fall in writhing response induced by an intraperitoneal injection of acetic acid (Fig. 5). When compared to the control, the HTI at the dose of 750 and 1000 mg/kg showed statistically significant (*P< 0.01) effects and the percentage inhibition of writhing with selected doses of the HTI were 44.4% and 48.9%, respectively, while standard drug diclofenac (25 mg/kg, *per oral*) showed 50.9% inhibition of writhing in experimental animals. The maximum anti-nociceptive activity was noted with higher dose (1000 mg/kg, *per oral*) which was comparable to the effect of the standard drug diclofenac (25 mg/kg, *per oral*).

### Membrane stabilization

**Fig. 6:**
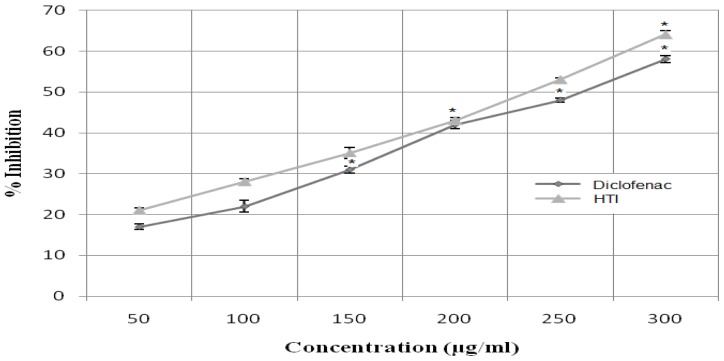
Effect of HTI and Percent inhibition of heat-induced hemolysis of rat erythrocytes. Each value was expressed as mean ± SEM (n=5). Experimental groups results were compared to those of the control (*P< 0.01).

The result showed that HTI at concentrations of 50–300 μg/ml defended the rat erythrocytes membrane against lysis induced by heat solution. Significant results (*P< 0.01) were found at concentrations higher than 200 μg/ml. The standard drug diclofenac offered significant (*P< 0.01) protection against damaging effect by heat solution at a concentration of 150 μg/ml (Fig. 6).

Pain and inflammation are associated with many pathophysiologic clinical conditions such as arthritis, cancer and vascular diseases [10]. A number of natural products are used in various traditional medical systems for the relief of symptoms associated with pain and inflammation. In the present study, we compared the effectiveness of *T. indica* leaves extract regarding antinociceptive and anti-inflammatory activity with the standard drug in treated and control animals, respectively. Our results revealed that HTI possessed significant antinociceptive activity in thermally and chemically induced rat pain models, and anti-inflammatory activity on carrageenan-induced rat hind paw oedema with membrane stabilizing effect through prevention of heat-induced lysis of RBC membrane.

Carrageenan-induced oedema is a multifaceted phenomenon that liberates a variety of chemical mediators and is believed to be a biphasic phenomenon [11]. The initial phase of oedema (0–1 h) is not inhibited by non-steroidal anti-inflammatory drugs (NSAIDs) and contributes to the release of histamine, serotonin and bradykinin [12, 13]. The second phase of oedema accelerates (1–5 h) swelling and is correlated with elevated production of TNF-α, NO, and PGs [14]. The second phase has contributed to the induction of cyclooxygenase-2 (COX-2) in the hind paw [15]. The synthesis of which is triggered by those cytokines, which also induces iNOS and free radicals. These substances are responsible for the formation of inflammatory exudates and inhibition of these chemical mediators from bringing out their pharmacological effects normally ameliorate the inflammation and other symptoms such as pain and fever [16, 17].

The results of the present study revealed that the administration of HTI inhibited the oedema starting from the first phase to second phase of inflammation. In this study, HTI showed an anti-inflammatory effect on carrageenan-induced rat paw oedema in both phases (0–4 h). Experimental results indicated that the anti-oedematic effects of HTI were due to the inhibition of neutrophils and TNF-α synthesis. Moreover, the anti-inflammatory action of HTI may be related to the inhibition of PGs and NO synthesis which is similar to the anti-inflammatory mechanism of diclofenac in carrageenan-induced inflammation [12]. The data showed that HTI possessed membrane stabilizing property, as it offered significant protection of RBC membrane against lysis induced by heat. The HTI also decreased the migration of WBC to the site of induced inflammation.

RBC membrane is similar to that of the lysosomal membrane; inhibition of RBC hemolysis therefore will provide good insights into the inflammatory process [18]. Injury to the lysosomal membrane usually triggers the release of phospholipase-A_2_ that mediates the hydrolysis of phospholipids to produce inflammatory mediators [19, 20]. Stabilization of the membrane of these cells inhibits lysis and subsequent release of the cytoplasmic contents which in turn limits the tissue damage and exacerbation of the inflammatory response [21]. It is therefore expected that compounds with membrane stabilization activity should offer significant protection of cell membranes against injurious substances. The *in vitro* ability of HTI to protect erythrocytes in opposition to heat-induced lysis in a dose-dependent approach indicated its capability to stabilize the lysosomal membrane and inhibited the inflammation induced by carrageenan.

Furthermore, plants with membrane-stabilizing properties are well known to interfere with the early phase of inflammatory mediator release, particularly by mediating the release of *phospholipase* A_2_ that triggers the formation of such inflammatory mediators [20]. It has been postulated that the abdominal writhing, the visceral or peripheral pain model, acts indirectly by inducing the release of endogenous mediators through cyclooxygenase (COX) as well as lipooxygenase pathway i.e. PGE_2_ and PGE_2α_ in peritoneal fluids, the processor releases arachidonic acid via COX and PGs biosynthesis. These mediators stimulate the nociceptive neurons which are sensitive to NSAIDs and related compounds [22–24]. In the present work, it was demonstrated that HTI and diclofenac caused a significant inhibition (48.9% and 50.9%, respectively) on the writhing responses induced by acetic acid as compared to the control group. This peripheral analgesic potential might be related to the inhibition of PGE_2_ and bradykinin, as we had also found that HTI reduced the oedema formation induced by these mediators which suggested that the HTI antinociceptive effect could be associated with an anti-inflammatory action.

To evaluate the role of HTI in the centrally mediated nociceptive pathway, we used the thermal model for nociception, the hot-plate test, a well-established model for the detection of analgesic activity of different types of drugs [25, 26]. The results of our study demonstrated that highest inhibition of nociception stimulus exhibited by HTI (1000 mg/kg, *per oral*) was observed after 120 min (7.12±0.05 sec) as compared to the control group. Hence the results of our study suggested that HTI might exert its analgesic effect in a way similar to that of pethidine by inhibiting the centrally mediated antinociceptive pathway or blocking the neural transmission of pain. The tail-flick response appeared as spinal reflex and was considered to be selective for centrally acting antinociceptive compounds [27]. This test is very useful for discriminating between centrally acting opiate-like analgesics and non-opiate analgesics. This test has been found to be suitable for the evaluation of centrally but not for peripherally acting drugs. The HTI showed antinociceptive activity in the tail-flick test. HTI at a dose of 1000 mg/kg increased the maximum latency time (36.06%) when tested at 60 min as compared to the control treated group. The pain latency time response was found to be similar to the effect of pethidine (4 mg/kg, *intraperitoneally*).

The results of the phytochemical screening revealed that HTI contained tannins, saponins, glycosides, flavonoids and polyphenols. The profile of polyphenols and flavonoids in *T. indica* has been reported previously for the presence of proanthocyanidine in various forms like apigenin, anthocyanin, procyanidine, catechin, epicatechin, along with taxifolin, eriodictyol and naringenin [28]. Out of these phytoconstituents, polyphenols and flavonoids have been well known to exhibit anti-inflammatory and antinociceptive action [29–31]. These flavonoids may interact directly with the prostaglandin system and inhibit the substitute cofactor for the prostaglandin generation and also inhibit arachidonate lipoxygenation as well as enzymes involved with inactivation or biotransformation of prostaglandins [32, 33]. In the present work, flavonoids were quantified. Thus, it could be concluded that flavonoids from *T. indica* might be responsible for the anti-inflammatory and antinociceptive potential.

## Experimental

### Preparation of hydroalcoholic T. indica leaves extract

The fresh leaves of *T. indica* were collected from tropical forest research area, Jabalpur in October 2010. The material was identified and authenticated by Dr. S. D. Upadhyaya, senior scientist and Botanist, Department of crop and herbal physiology, Jawaharlal Nehru Krishi Vishwavidyalaya, Jabalpur, M.P., INDIA and the voucher specimen number HD/CHPY/9581 was deposited. The leaves were air-dried and made into powder with the aid of local mortar and pestle. Extract was prepared by taking 100 g of *T. indica* leaf powder and soaking it in solvent consisting of 95% of ethanol and water (1:1) for 72 h. The residue was filtered by whatman filter No. 4, and the extraction solvent was removed under reduced pressure by rotary vacuum evaporator (Superfit, India) at 50 °C, which gave a reddish-brown residue. Then this hydroalcoholic leaves extract (HTI) was freeze dried and stored at 4 °C until use.

### Phytoanalytical studies

#### HPLC analysis

HPLC analysis was performed by following the previously reported method with slight modification [34]. In brief, for this purpose Shimadzu LC-20A apparatus coupled with a semiautomatic injector (SIL-20A, Shimadzu) and UV/VIS detector (SPD-20A, Shimadzu), and spin-chrome CFR software was used. A Shim-pack ODS column luna C-18 (5 μm, 4.6/250 mm; Shimadzu) was employed coupled to the respective guard-column, using the following elution profile: 0–10 min: 2% B (isocratic), 10–100 min: 2–20% B (linear gradient), 90–120 min: 20–100% B (linear gradient); solvents: A = aqueous acetic acid 2% (v/v); B = MeCN with 2% acetic acid (v/v). Flow rate: 1 ml/min. UV detection between 200 and 750 nm.

#### Preliminary phytochemical screening

The extract was subjected to preliminary qualitative test to identify various phyto-constituents present in the leaves [35]. Results of phytochemical screening revealed that HTI contained tannins, saponins, steroids, carbohydrates, a fraction of flavonoid, isoflavonoid and polyphenolic compounds.

#### Determination of phenolics

The total phenolic content of extract was determined as per the reported Folin–Ciocalteu method [36]. In brief, 200 μl of diluted extract was added to a test tube and then mixed with 500 μl of Folin Ciocalteu reagent (1:10). Thirty seconds later and just prior to 8 min, 800 μl of Na_2_CO_3_ (7.5%) was added. The reaction mixture was incubated at 24 °C and absorbance of mixtures was traced at 750 nm against blank. The standard curve was prepared by 1, 10, 100 and 200 mg/l solutions of gallic acid in ethanol: water (50:50 v/v) solvent. The values of total phenolic were estimated by comparing the absorbance of each with those of a standard response curve generated with gallic acid and the total phenolic content was expressed as gallic acid equivalents per mg of HTI (Table 1).

#### Determination of total flavonoids

The concentrated extract was again exhaustively defatted by refluxing with *n*-hexane and benzene (twice for 15 h). HTI (0.5 ml of 1:10 g/ml) in ethanol was separately mixed with 1.5 ml of ethanol, 0.1ml of 10% aluminum chloride, 0.1 ml of 1 M potassium acetate and 2.8 ml of distilled water. It remained at room temperature for 30 min. A dark yellow color indicated the presence of flavonoids [37]. The absorbance of the reaction mixture was measured at 415 nm (Table 1).

### Acute toxicity study

For the purpose of acute toxicity study of HTI, Specific-pathogen-free healthy male Wistar albino rats were obtained (Regional Cancer Research Institute of Gwalior, Madhya Pradesh) and acclimatized to the laboratory conditions (R.N.S. College of Pharmacy, Gormi Bhind, M.P.). They were housed under conventional conditions in polypropylene cages in a controlled environment. They were provided with water and food to acclimatize them before starting the experiment. Experimental protocols were approved by the institutional ethical committee (Regd. No. 1030/a/07/CPCSEA). The selected animals were divided in 4 groups and each group consists of 5 male Wistar albino rats. The animals of I, II and III group were fed orally with HTI in increasing dose levels of 500, 750 and 1000 mg/kg body weight, respectively while control group received normal saline 2 ml/kg. The animals were observed continuously for 2, 4 and 6 h after administration for their behavioral, neurological and autonomic profile. No death was observed at highest dose. The intensive observations were taken for 7 days. After the treatment period of 7 days, all animals were anaesthetized for blood collection by cardiac puncture. Blood samples were analyzed for different hematological parameters. Blood cell count was done with blood smears and hemogram was performed on ACT diff-2 Hematology Analyzer (Beckman Coulter India, Ltd., Mumbai, India) [38].

### Experimental animal and group design

In the present study healthy male Wistar albino rats (150–200 g) were selected. They were housed as per standard environmental conditions (12:12 h light/dark cycle; 25±3°C; 40–60% humidity). Eighteen hours before experiment, food was withdrawn but water remained *ad libitum*. All experimental protocols were approved by Institutional Animal Ethical Committee of the Institute (Regd. No. 1030/a/07/CPCSEA). Each study has individual group of animals during the whole experiment and all animals were divided into five groups (n=5). Group I served as control and received normal saline (2 ml/kg, *per oral*), group II served as Standard drug treated and received diclofenac (25 mg/kg, *per oral*) and pethidine (4 mg/kg, *intra peritoneal*) as per selected model, group III, IV and V served as test drug treated and received HTI at the dose of 500, 750 and 1000 mg/kg, *per oral*, respectively.

### Carrageenan-induced rat paw oedema

Carrageenan-induced paw inflammation was produced and the volume of the right hind paw of each rat measured using a plethysmometer (Orchid Scientific & Innovative Pvt Ltd, India) according to the standard methods with certain modifications [39]. After 1 h of administration of standard and test drug, 0.1 ml (1% w/v) λ-carrageenan in normal saline was injected subcutaneously into the sub-planter region of right hind paw under mild ether anesthesia using a 25 G needle and 1 ml syringe. The measurement of paw volume was accomplished immediately by mercury displacement technique using the plethysmometer at hourly interval up to 4 h. The degree of swelling was calculated by the paw volume increase (V_t_−V_0_) where V_t_ and V_0_ are the volume of the left hind paw after and before the carrageenan injection, respectively. The percent inhibition of inflammation at each h compared to the controls was calculated for each group as follows:

$$\text{Percent inhibition}=\frac{{\left({\text{V}}_{\text{t}}-{\text{V}}_{0}\right)}_{\text{in control rats}}-{\left({\text{V}}_{\text{t}}-{\text{V}}_{0}\right)}_{\text{in treated rats}}}{{\left({\text{V}}_{\text{t}}-{\text{V}}_{0}\right)}_{\text{in control rats}}}\times 100$$

### Eddy’s hot plate method

Animals of separate groups received HTI (500, 750, and 1000 mg/kg) and normal saline (2 ml/kg) orally. Pethidine (4 mg/kg, intra peritoneal) was used as a standard drug. The animals were individually placed on the hot plate maintained at 55±1°C, 1 h after their respective drug treatments. The response time was noted as the time at which animals reacted to the pain stimulus either by paw licking or jump response, whichever appeared first. The cutoff time for the reaction was 15 s. The reaction time in control and treated group was recorded as 0, 30, 60, 120 and 180 min after the treatment [40].

### Tail flick method

Animals of separate groups received HTI (500, 750, and 1000 mg/kg) and normal saline (2 ml/kg) orally. Pethidine (4 mg/kg, intra peritoneal) was used as a standard drug. After 15 min of administrations of test and standard drug the antinociceptive activity was measured. Briefly, the tail of the rat was placed on nichrome wire of analgesiometer (Techno, India) and the time taken by the animal to withdraw (flick) its tail from the hot wire was taken as the reaction time and was recorded at 15, 30, 60, 90 s time interval after the drug administration, The cutoff time for the reaction was 15 s [41].

### Acetic acid-induced writhing response

Acetic acid-induced writhing response was measured as per the previously reported method with slight modifications [24]. Abdominal muscle contractions were induced in rats by intraperitoneal injection of 0.6% solution of acetic acid (10 ml/kg). Thirty minutes prior to this administration, the animals were treated orally with diclofenac (25 mg/kg) and HTI (500, 750 and 1000 mg/kg), respectively. The number of writhes (abdominal muscle constrictions accompanied by stretching of hind limbs) occurring between 5 and 20 min after acetic acid injection was counted.

$$\%\text{inhibition of writhing}=\frac{\text{Writhing of}\;\text{control group}-\text{Writhing of test group}}{\text{Writhing of control group}}\times 100$$

### Membrane stabilizing activity

*In-vitro* heat-induced hemolysis of rat erythrocytes associated with membrane stabilizing activity of a test drug was evaluated as per the reported method [42]. Blood was collected from the tail tip of the rat under mild ether anaesthesia taking aseptic precautions. Briefly, a vial containing 20 μl fresh rat blood in 2 ml of Phosphate Buffer Saline (PBS) was treated in triplicate with the HTI or diclofenac (the standard drug) so that the final concentration of the HTI or diclofenac in the vials became 50, 100, 150, 200, 250 and 300 μg/ml. Each control vial contained 20 μL of PBS. The vials were incubated for 15 min at 37 °C followed by 54 °C for 15 min then centrifuged it and absorbance of supernatant was measured spectrophotometrically at 540 nm. The percent inhibition of haemolysis was calculated as:

$$\text{Percent inhibition}=\frac{{\text{A}}_{\text{control}}-{\text{A}}_{\text{sample}}}{{\text{A}}_{\text{control}}}\times 100$$

The EC_50_ value of the HTI and diclofenac for the plasma membrane stabilization effect was calculated using the plot of concentration of the HTI or diclofenac versus percent inhibition of hemolysis.

### Statistical analysis of data

Data are given as means ± SEM, (n=5). Significant differences between groups were determined by analysis of variance (ANOVA) complemented with Dunnett’s post hoc multiple range tests where the *P* value ≤ 0.01 was considered as significant.

## Figures and Tables

**Tab. 1:** Flavonoids and Total Phenolic contents of HTI

Extract	Flavonoids (mg/g)	Polyphenols (mg/g)
Hydroalcoholic extract of *T. indica* leaves (HTI)	26.12 ± 2.54	53 ± 3.21

Values were expressed as mean ± S.E.M. (n=3).

**Tab. 2:** Effect of oral administration of HTI on neurobehavioral profile of Wistar rats

Effect	Response
Death	−
Convulsions	−
Tremor	−
Rigid tail	−
Sedation	+
Excitation	−
Jumping	++
Abnormal gait	−
Motor in-coordination	−
Altered muscle tone	−
Loss of grasping	−
Akinesia	−
Catalepsy	+
Loss of traction	−
Writhing	−
Stereotypy	−
Head twitches	−
Scratching	−
Altered respiration	−
Aggression	−
Altered fear	−
Altered reactivity to touch	−
Ptosis	−
Exophthalmia	−
Loss of righting reflex	−
Loss of corneal reflex	−
Analgesia	++
Defecation	−
Salivation	−
Lacrymation	−

+…slight; ++…moderate; +++…high; −…no response.

**Tab. 3:** Effect of orally administered HTI on hematological parameters of male Wistar rats

Parameters	Control (2 ml/kg)	HTI (500 mg/kg)	HTI (750 mg/kg)	HTI (1000 mg/kg)
RBC_s_ (millions/cu.mm)	5.12 ± 0.40	5.16 ± 0.35	5.27 ± 0.37	5.33 ± 0.38
Hg (%)	13 ± 0.28	13.81± 0.98	14.1 ± 1.1	14.11±1.0
PCV (%)	42.11 ± 1.05	44.5 ± 4.1	44.0 ± 2.5	45 ± 1.1*
WBC_s_ (cells/cu.mm)	7369 ± 435.13	8041 ± 328.9**	8320 ± 409.3*	8569 ± 522.3*
Neutrophils (%)	53.61 ± 4.79	43 ± 2.8**	46± 3.3*	48 ± 3.7*
Lymphocytes (%)	39.01 ± 1. 42	47 ± 3.2**	48 ± 3.9	46 ± 4.1*
Eosinophils (%)	7.0 ± 0.48	5.0 ± 0.8*	5 ± 0.4*	5 ± 0.3*
Monocytes (%)	4.0 ± 0.02	3.0 ± 0.24	4.0 ± 0.3	2 ± 0.1*
Basophils (%)	0 ± 0	0 ± 0	0 ± 0	0 ± 0
Platelets count (10^5^ cells/cu.mm)	1.52 ± 0.05	1.86 ± 0.09*	1.89 ± 0.12*	2.1 ± 0.14*
MCV (F1/red cells)	78.5 ± 2.6	80.8 ± 1.6	83.5 ± 2.4*	84.6 ± 5.6*
MCHC	27.3 ± 1.6	24.91 ± 1.1	29.6 ± 1.3	30.15 ± 2.3*

PCV (Packed cell volume); RBC (Red blood cells); WBC (White blood cells); Hg (Haemoglobin); MCV (Mean corpuscular volume); MCHC (Mean corpuscular hemoglobin concentration). Results were expressed as mean ± S.E.M (n=5). The *P< 0.01 and **P< 0.001 value was found to be significant as compared to the control group.
